# Polymeric Micro Sensors and Actuators

**DOI:** 10.3390/s140815065

**Published:** 2014-08-15

**Authors:** Wei-Chih Wang

**Affiliations:** Department of Mechanical Engineering, University of Washington, Seattle, WA 98185, USA; E-Mail: abong@u.washington.edu; Tel.: +1-206-543-2479; Fax: +1-206-685-8047

Sensors and actuators using polymeric systems, constitute one of the most promising fields of “smart polymers”, and it is becoming ever more important to associate artificial sensing and actuating systems with living organisms. Testing of some practical applications has now started in industry. The aim of this Special Issue is to develop a basic understanding of the field, its supporting technologies and current applications. The special issue has collected interesting papers covering topics dedicated to a wide range of applications, in particular in the areas of humidity [1,2], chemical [3], mechanical [4,5], electromagnetic [6], electrochemical, piezoelectric [1,3,6], biosensors [3,7] and wearable sensors and actuators. In addition, the issue has also received papers from different scientific groups regarding methods in fabrication [8], materials characterization [5,9] for optimization of polymer sensors and actuators performances, as well as supporting electronics [2]. All accepted papers represent a stimulus for all of us since they bring us interesting novelties for encouraging the audience to continue research in the right directions. Papers that have not been accepted also have merit for having helped this Editor and Reviewers extract useful information. Most of them, even if rejected, take different approaches perhaps not suitable for this journal, but surely appropriate for other reputed publications, so we take advantage for this Editorial to thank them. Finally, we would like to thank the Editor-in-Chief and the *Sensors* journal staff for their support to produce this special issue and to handle the manuscript review process.
